# A motion compensation treadmill for untethered wood ants (*Formica rufa*): evidence for transfer of orientation memories from free-walking training

**DOI:** 10.1242/jeb.228601

**Published:** 2020-12-22

**Authors:** Roman Goulard, Cornelia Buehlmann, Jeremy E. Niven, Paul Graham, Barbara Webb

**Affiliations:** 1 School of Informatics, University of Edinburgh, Edinburgh EH8 9AB, UK; 2 University of Sussex, School of Life Sciences, Brighton BN1 9QG, UK

**Keywords:** Insects, Navigation, Visual memory, Associative learning, Motion compensator, Treadmill, Untethered

## Abstract

The natural scale of insect navigation during foraging makes it challenging to study under controlled conditions. Virtual reality and trackball setups have offered experimental control over visual environments while studying tethered insects, but potential limitations and confounds introduced by tethering motivates the development of alternative untethered solutions. In this paper, we validate the use of a motion compensator (or ‘treadmill’) to study visually driven behaviour of freely moving wood ants (*Formica rufa*). We show how this setup allows naturalistic walking behaviour and preserves foraging motivation over long time frames. Furthermore, we show that ants are able to transfer associative and navigational memories from classical maze and arena contexts to our treadmill. Thus, we demonstrate the possibility to study navigational behaviour over ecologically relevant durations (and virtual distances) in precisely controlled environments, bridging the gap between natural and highly controlled laboratory experiments.

## Abstract (precis)

**Summary:** We have developed and validated a motion compensating treadmill for wood ants which opens new perspectives to study insect navigation behaviour in a fully controlled manner over ecologically relevant durations.

## INTRODUCTION

Social insects often need to forage for sparsely distributed food sources in extended complex environments. To achieve this, they utilize efficient navigation strategies, travelling long distances to find food to bring back to the nest (ants: Collett and Collett, 2002; Cheng et al., 2014; Knaden and Graham, 2016; bees: Menzel et al., 2005). Field studies and laboratory experiments have been used to investigate the underlying mechanisms of sensorimotor control and memory that drive such efficient navigation, but have inherent limitations. For instance, detailed observations of an animal's path are only possible over short distances and for short durations, compared to natural foraging. Furthermore, identification of sensorimotor transformations, as for any reverse engineering method (Villaverde and Banga, 2014), needs precise control or observation of the coupling between the output (behaviour) and the input (sensory stimulation), which is difficult in the field because of its large spatial extent and its heterogeneity (Nagaya et al., 2017). As an alternative, virtual reality (VR) methods provide a complementary tool (Schultheiss et al., 2017) to systematically control the access to sensory information for an individual while simultaneously recording precise behavioural responses, potentially over durations equivalent to long-distance foraging.

One commonly used apparatus for establishing VR with walking insects is an air- or bearing-suspended trackball (Schultheiss et al., 2017) or wheels (Hedwig, 2017) driven by a tethered animal. However, tethering will inevitably induce physical discrepancies in the sensorimotor control of behaviour (Paulk et al., 2015; Stowers et al., 2017), potentially affecting the quantitative identification of sensorimotor transformation(s) from the analyses of input–output relationships. In addition, the constant constraints applied, both by the tether and by the inherent manipulations to set it, can quickly induce aversive experiences and subsequently change the behaviour of ants engaged in a navigation task (Wystrach et al., 2020). It also reduces the ease of transferring animals between the real world and VR, e.g. for interleaved training and testing. Long-term fixing of a tether can be a problem for insects such as ants that need to deal with narrow tunnels in their nest, although this has been partly addressed using magnetic paint on the animal as the means of fixing them to an external tether Dahmen et al. (2017). Finally, a tether might directly constrain or prevent some aspects of the behaviour that we might wish to observe, such as the details of gaze control or posture, when a subject explores a visual scene. As a consequence, it is highly desirable to have a VR setup in which navigating insects can walk and behave freely (Stowers et al., 2014, 2017).

In this paper, we adapt for use in wood ants (*Formica rufa*) a motion compensator setup, previously proposed to study freely moving beetles (Götz and Gambke, 1968), honeybees (Kramer, 1976), crickets (Weber et al., 1981) and spiders (Stowers et al., 2014). This type of setup has been more recently modified to study olfactory guided behaviour in moths (Shigaki et al., 2016), demonstrating its efficacy. The general approach of ‘motion compensation’ is to have the animal walking, untethered, on top of a servosphere, and to use a camera to detect its motion and then to move the sphere beneath the animal to compensate. The latencies of early motion compensation systems, e.g. at around 150–400 ms (Kramer, 1976), may have led to erroneous conclusions (Schmitz et al., 1982) and limited the popularity of this approach. However, current technology, such as high-speed cameras and efficient microcontrollers, enable the implementation of full compensatory closed loop control with high temporal resolution (Shigaki et al., 2016). Using a motion compensator system for robust control of insect position has mostly been used for analysis of taxis behaviours guided by sound (Weber et al., 1981; Schmitz et al., 1982), olfaction (Kramer, 1976; Sakuma, 2002; Shigaki et al., 2016) or vision (Stowers et al., 2014), but it has significant potential for the study of more complex navigation behaviours.

Our aim is to demonstrate that it is plausible to use a motion compensation treadmill to observe naturalistic behaviour of untethered navigating wood ants, to gain mechanistic insight into their visual navigation. Wood ants are a good model to study visual memory and navigation because they have been shown to produce reliable navigation in indoor laboratory experiments (Durier et al., 2003; Graham et al., 2003). We demonstrate memory transfer from traditional conditioning experiments and landmark navigation experiments to the treadmill apparatus. Furthermore, we show that disturbances created through the control of the servosphere (e.g. vibration or jitter) do not alter ant movement statistics, and we show, with trials of up to 2 h, that ants remain motivated to walk in the conditions experienced on the treadmill. The results demonstrate the potential of the treadmill system for studying naturalistic orientation behaviours.

## MATERIALS AND METHODS

### Motion compensation treadmill

**Fig. 1.**
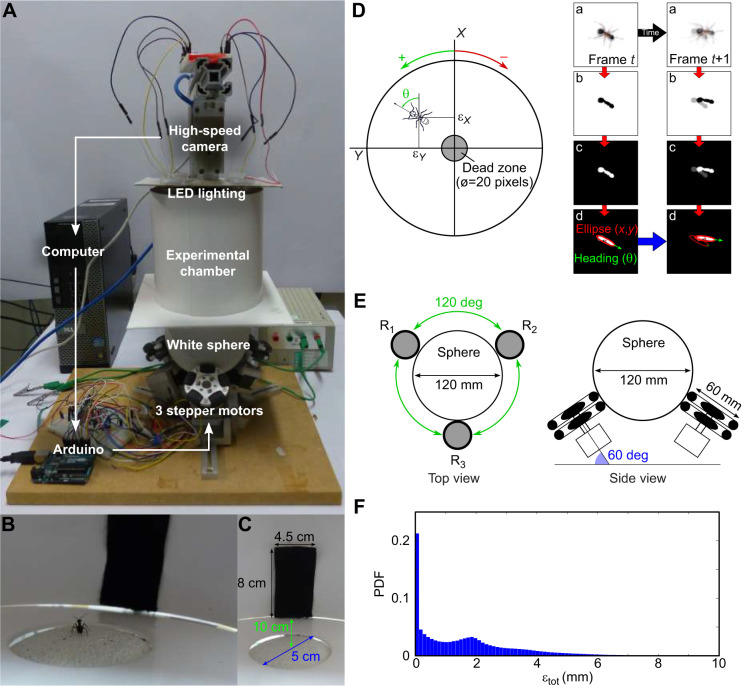
**Treadmill for freely moving ants.** (A) The motion compensator experimental set-up. (B) View from inside the experimental chamber showing an ant facing the cue. (C) The black fabric used as a visual cue during the experiments. (D) The tracking program extracts the position (ε*_X_*, ε*_Y_*) and orientation (θ) of the ant on the servosphere. The orientation is tracked with an uncertainty of ±180 deg, which is corrected afterwards assuming ants mostly move forward. The left panels show the different stages of the tracking procedure: acquisition of an optically blurred image (a), binarization (b), contrast inversion (c) and ellipse detection (d). Tracking consistency between two consecutive frames is ensure by minimizing the centroid speed (Δ*_XY_*) and the heading change (Δ_θ_). Only the *X* and *Y* positions are used to monitor the servosphere (see Fig. 2). (E) Top and side view from the rotors and servosphere system. The azimuth (120 deg) and the elevation (60 deg) angle are used to compute the rotational matrix used to transform the rotation of the sphere into the rotors reference frame (Eqn 4). (F) Probability density function (PDF) of errors from the centre measured by the tracking program along a batch of 174 experiments (10 to 15 min each).

The treadmill (Fig. 1A) is based on a setup previously proposed to study moth olfactory responses (Shigaki et al., 2016). Ants walk on a white 3-DOF (degrees of freedom) servosphere (foam, diameter: 120 mm) supported by three rotors (stepper motors, SANYO SY42STH38 - 0406A) underneath, each one equipped with a dual-disc omniwheel (aluminium, diameter: 60 mm, 2 discs, 5 rollers/disc) to avoid creating friction when the ball has to rotate perpendicularly to the axis of the rotor. Rotors are controlled using an Arduino Uno connected via USB to a computer (running Ubuntu 17) and three motor drivers (STMicroelectronics, ULN-2064B). The rotation of the ball is controlled by the ant's motion, which is tracked by a high-speed zenithal camera above the sphere. The camera is placed 150 mm above the top of the sphere and we used a 12 mm focal lens objective. The servosphere is lit from above by four lights, each consisting of three white LEDs and a diffusive cover to produce homogeneous lighting to facilitate tracking. A homogeneous cardboard ceiling, holding the LED lights, also blocks visual cues from above the setup. A white board, with a 5 cm diameter hole to let only the top of the servosphere pass (Fig. 1D), covers the bottom of the setup, and a circular barrier of white flexible plastic encloses the experimental chamber (Fig. 1A) and offers a support to set visual cues (Fig. 1D).

**Fig. 2.**
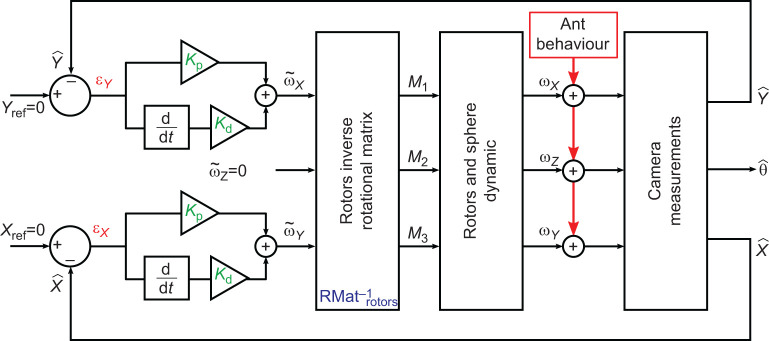
**Block diagram of the complete servosphere proportional–derivative control.** The coordinates of the ant (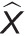 and 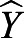) are used to calculate the errors ε*_X_* and ε*_Y_* from the centre of the ball (*X*_ref_ and *Y*_ref_ equal to 0) along the *X*- and *Y-*axes, respectively. Two separate proportional–derivative controllers (set with a proportional gain *K*_p_, and a derivative gain *K*_d_) are used to calculate the two theoretic corrective servosphere speeds (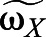,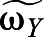) in addition to 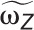 being kept at zero. These three values are transformed into the three rotor reference frames to obtain the signal (*M*_1_, *M*_2_, *M*_3_) that will generate the motion compensation (ω*_X_*, ω*_Y_*, ω*_Z_*). The camera therefore ensures the new measure of the ant coordinate (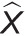 and 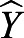) as well as its body orientation (θ).

#### Tracking the ant

The position and [−90; 90] deg orientation of the ant are tracked using a high-speed camera (Basler ace acA640-750 μm – monochrome, 700 frames s^−1^) and a custom Python program. Ants are detected by binarizing the frame, using a threshold (set by the experimenter to lower background noise) and reversing the contrast, to make ants appear as a white ‘blob’ on a black background (Fig. 1B). Then, contours are extracted from which we select the blob most likely to be the ant, based on approximate size and minimization of the distance to the previously detected location. From the selected blob, we extract the position of the centroid and the [−90; 90] deg orientation by approximating an ellipse (facilitated by the blurring from the unfocused camera lens). The heading of the ant is approximated with a ±180 deg uncertainty, which is resolved in post-processing by assuming the ant moves forwards, and kept consistent by minimizing the heading change between two consecutive frames (Fig. 1). As a consequence, the ±180 deg confusion persists when the ant is not moving, so subsequent analysis involving orientation excludes periods of inactivity.

#### Moving the 3-DOF servosphere

The *X* and *Y* distances from the centre are used to actuate the rotation of the sphere and keep the ant at the centre of the treadmill. In this work, we only controlled the position of the ant on the treadmill and let it freely rotate in a closed-loop manner. The extracted coordinates of the ant, 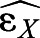 and 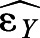 in pixels, are used to drive two independent proportional–derivative controllers to define the proper angular speed to apply to the ball (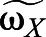,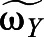). Both controllers are set with the same parameters and 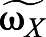 and 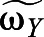 are calculated as follows:(1)
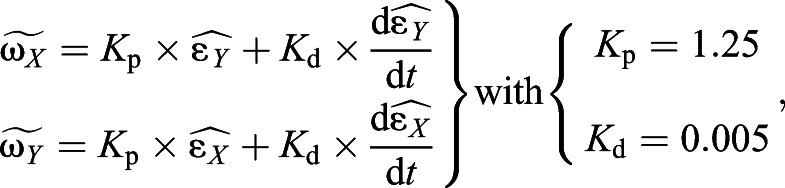


where *K*_p_ is the proportional gain, *K*_d_ is the derivative gain and *t* is time. Then, ω*_X_* and ω*_Y_* are transformed into the reference frame of the three rotors to define the motor input necessary to actuate the sphere properly. The transformation matrix is computed using the azimuth and elevation of the rotors' axes of rotation:(2)
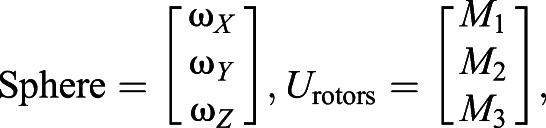
(3)
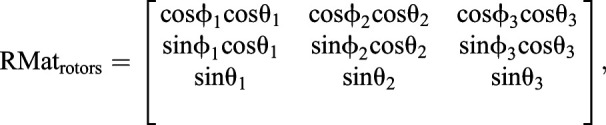


where ω*_X_*_,*Y*,*Z*_ is the actual angular speed of the servosphere, *M*_1,2,3_ is the motor speed of rotation, φ_1,2,3_ is the elevation angle from the horizontal plane of the rotors' axis of rotation and θ_1,2,3_ is the azimuth angle, i.e. the orientation in the horizontal plane. *U*_rotors_ is the matrix of the control sent to the rotors and RMat_rotors_ is the rotation matrix of the rotors. Therefore, the control signal to send to the rotors can be extracted from the kinematics of the sphere:(4)
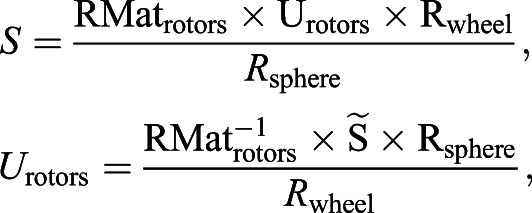


where *S* is the actual dynamics of the sphere, 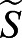 is the desired dynamics of the sphere, *R*_wheel_ is the radius of the omniwheels and *R*_sphere_ is the radius of the sphere. From Eqn 4 it is determined what motor inputs will drive the sphere at the determined angular speeds (after the calculation of 
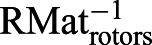
, Eqn 6):(5)
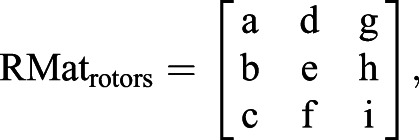
(6)
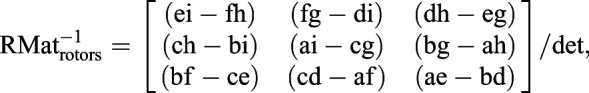
(7)
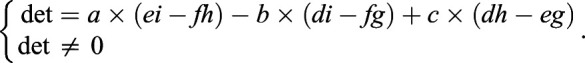


For the present setup, rotors are placed equally around the ball on the *X*–*Y* plane (θ_1_=0, θ_2_=120 and θ_3_=–120 deg) and oriented around 60 deg from the horizontal (φ_1,2,3_=60 deg) (see Fig. 1C). The three rotor inputs are sent to the Arduino board via serial communication. The resulting feedback loop to control the treadmill is maintained at a frequency of around 500 Hz. The full system is represented in the block diagram presented in Fig. 2.

### Insects

Colonies of queen-right wood ants (*Formica rufa* Linnaeus 1761) were collected from woodland (Broadstone Warren, East Sussex, UK) and housed in large tanks in the laboratory in the University of Sussex at 20–25°C. Water, sucrose and dead crickets were provided *ad libitum* on the surface of the nest. To increase the ants' foraging motivation, food was kept at a minimum during the experiments, but ants had access to water all the time.

### Testing innate behaviours

To evaluate any effects of the treadmill on the movement pattern and motivational state of ants, we conducted a series of experiments in simple environments. First, we surrounded the servosphere with a homogeneous white wall 10 cm from the centre of the sphere. We measured two main characteristics of the ants' paths: the transition rates between active and inactive phases of movement; and the turning rate, represented by the angular speed. The statistics measured on the treadmill were compared with statistics measured from ants exploring a 12 cm diameter circular arena surrounded by a uniform white wall. Secondly, we added a black vertical bar to the environment, to trigger directed behaviour towards the single conspicuous cue as previously observed in many insect species (Wallace, 1962; Voss, 1967; Wehner, 1972; Götz, 1987; Collett, 1988; Graham et al., 2003; Poehlmann et al., 2018 preprint; Giraldo et al., 2018; Green et al., 2019).

### Testing associative learning

**Fig. 3.**
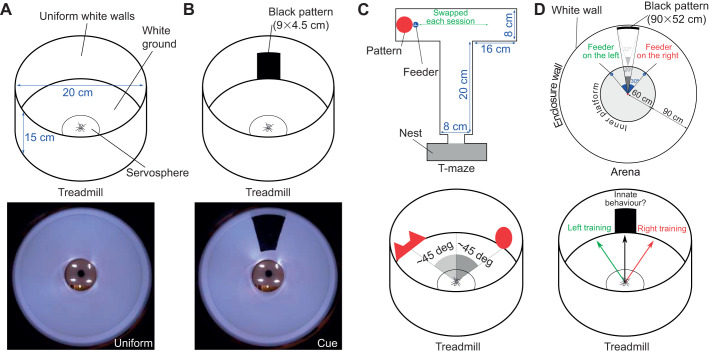
**Schematic of the behavioural protocols.** (A) Innate movement patterns were recorded on the treadmill in a circular arena of 20 cm diameter and 15 cm height. The picture at the bottom shows the visual environment from the ants' point of view on the servosphere. (B) Innate response to a visual cue (a vertical black bar) was tested on the treadmill. The picture at the bottom shows the environment with the visual cue from the ants' perspective. (C) Protocol used to test transfer of associative learning onto the treadmill. Upper panel: circle pattern was reinforced by systematically associating it with food reward in a simple T-maze. The pattern was placed in a different arm of the maze for each training session. Lower panel: preference for the pattern was tested in competition to assess the effect of the reinforcement. The patterns were always presented at the same location with respect to the centre of the servosphere. (D) Protocol used to test the transfer of navigational memory onto the treadmill. Upper panel: a subset of ants were trained in an arena (150 cm radius) to find a feeder located 30 deg to the right or left of the outside of a black rectangle placed on the white circular wall of the arena. Lower panel: ants that reliably reached the feeder in the arena were tested on the treadmill for around 10 min.

Twelve ants were tested (so-called pre-training group) on the treadmill to evaluate their innate preference for two different patterns made of red cardboard: a red circle, and two triangles merged together (Fig. 3C). Then, training was conducted in a T-maze connected to the ants' nest-box (including individuals from the pre-training group and additional ants naive to these patterns). At the end of one arm of the T-maze we placed the circle pattern along with a feeder (50% water, 50% sugar). The arm in which it was placed was switched for every training session. Each session consisted of 12 h free exploration of the T-maze, after which the ants were collected and either tested or moved back into the nest-box. Ants had no access to food between each exploration session, to maintain their motivation. Post-training ants were tested on the treadmill using both patterns at once, in a competitive manner. The position of each pattern was kept constant during the trials on the treadmill (circle at −45 deg, two triangles at +45 deg; Fig. 3C). The post-training group included pre-tested ants and ants that had never experienced the treadmill. We did not have information for individual ants about their degree of training in the T-maze. Nevertheless, each tested ant had been observed to leave the nest-box during at least one training session, and therefore had potentially explored the complete T-maze.

### Navigational task

Active foragers were selected based on their motivation to approach a small cylinder placed around 20 cm from a release point (i.e. only ants that directly moved toward the cylinder and reached it were selected for the experiment). These ants were individually marked (to be tracked across training and test sessions) and then trained in an arena to find a feeder displaced at a constant angle from a single, more distant, visual cue (Fig. 3D). The arena consisted of a 120 cm diameter platform surrounded by a white cylinder of 300 cm diameter and 180 cm height. The visual cue was a black rectangle (52 cm wide, 90 cm high) placed on the outer wall. Throughout the training protocol, a feeder (67% water, 33% sugar) was placed at the outer edge of the central platform, offset by 30 deg to the right or left of the outside edge of the black rectangle. Each marked ant participated in several training sessions, initially in groups of five to six ants, progressing to individual training. Ants were released in the centre of the central platform at the beginning of each session and allowed to explore until they reached the feeder, where they could feed *ad libitum*. After they exited the feeder, ants had a short amount of time to further explore the surroundings before being returned to the nest, where they could express trophallaxis with the rest of the colony. Marked ants showing foraging activity on the surface of the nest would be collected later for a new training session. In total, including both collective and individual training, a training day included ∼10–15 sessions. After 2 to 3 days of training, the best trained ants, characterized by direct paths to the feeder on at least three sessions in a row, were selected to be tested on the treadmill.

On the treadmill, ants were tested with a vertical bar of 4.5 cm width and 8 cm height placed 10 cm away from the centre of the servosphere (25×38.5 deg), which matches approximately the angular size of the cue experienced by the ants near the start of their trip from the centre of the arena (20×31 deg) to the edge of the platform (60×71.5 deg). The visual cues used in both contexts have the same width/height ratio (treadmill: 0.5625; arena training: 0.5778). Each selected ant was tested once for 10 min, after which it was placed in a tank providing sugar and then moved back to their nest, where they could provide food to the rest of the colony through trophallaxis. Some ants previously tested on the treadmill were later collected (when showing foraging activity at the surface of the nest) to undergo a new ‘training’ session in the familiar arena. This was to check for any change in their motivation to forage or extinction of their visual memory owing to the testing on the treadmill.

## RESULTS

### Innate behaviours

One of our aims in developing an untethered treadmill for ants was to avoid artefacts caused by tethering and, hence, to be able to observe robust naturalistic behaviour in virtual reality. However, the potential vibration produced by controlling the 3-DOF servosphere could also cause some disturbance that modifies the ants' behaviour. In order to measure the consistency of ant movements, we conducted a set of experiments to quantify innate behaviour on the treadmill in comparison to conventional arena experiments.

**Fig. 4.**
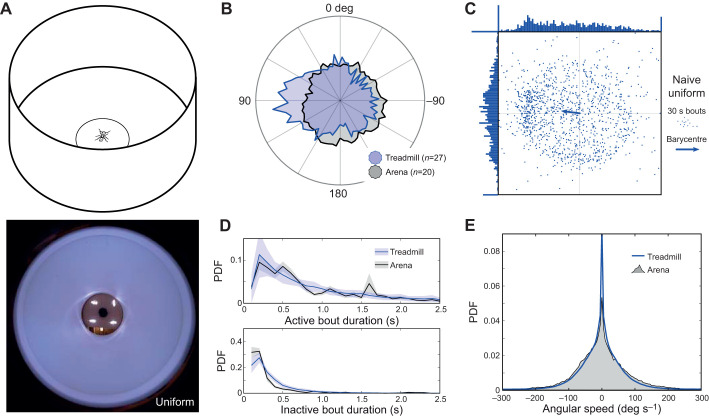
**Walking in a homogeneous white environment on the treadmill compared** **with** **arena behaviour.** (A) Conservation of ants' innate motor patterns was tested on the treadmill in a circular arena of 20 cm diameter and 15 cm height. Lower panel shows the visual environment from the ants' point of view on the servosphere. (B) Probability density function of the heading measured on the treadmill (blue patch) and in an arena (black patch), both in a uniformly white environment. The distribution of headings onto the treadmill shows a statistically significant bias from uniformity (Hodges–Ajne test: arena, *n*=20, *z*=4, *P*=0.11; treadmill, *n*=27, *z*=5, *P*=0.020). (C) Mean resultant vector extracted from the experiment in a uniformly white environment on the treadmill. Each point represents the mean resultant vector of movement for a 30 s bout (i.e. vectorial sum of all the unitary movements during 30 s). The arrow indicates the overall resultant vector, showing there is a small bias, mostly along the *Y*-axis, in the uniform arena (omnibus test, *n*=27, *z*=5, *P*=0.020). (D) Distribution of active and inactive bout length during the experiments on the treadmill (blue line) and in the arena (black line) in a uniformly white environment. Shading indicates ±s.e.m. (E) Distribution of angular speed measured during the experiments on the treadmill (blue line) and in the arena (black patch) in a uniformly white environment.

First, we placed ants in a uniform environment to measure motion statistics on the compensator and in an arena (Fig. 4). Wood ants showed a robust exploratory behaviour during long periods of time on the treadmill, including many 15 to 30 min bouts without significant drops in activity, and one ant running for 150 min. Ants surrounded by a uniform white environment (Fig. 3B) explored the entire extent of the virtual arena, although they expressed a slight preference towards one direction (Hodges–Ajne test: *z*=5, *P*=0.020; Fig. 4B). To look in more detail at the movement of ants on the treadmill, we selected two main parameters of interest. The first parameter we measured represents the tendency of wood ants to move in a stop-and-go manner. We measured the distribution of the lengths of each of the walking (active) and pausing (inactive) bouts. We observed a fairly similar distribution between arena and treadmill experiments in terms of active and inactive bout length (Fig. 4C, Fig. S1BC), showing that this movement pattern is not affected by our setup. The second parameter we examined is the distribution of angular speeds, as a turning-rate proxy. On both the treadmill and in an arena, ants displayed the same range of angular speed between –200 and 200 deg s^−1^ (Fig. 4D, Fig. S1A), although we observed an increase in the peak at 0 deg s^−1^ on the treadmill, i.e. a higher tendency to walk straighter. The real cause of this difference is unexplained, though it may arise from uncontrolled directional cues, which could also have induced the general direction bias we observed (Fig. 4C).

**Fig. 5.**
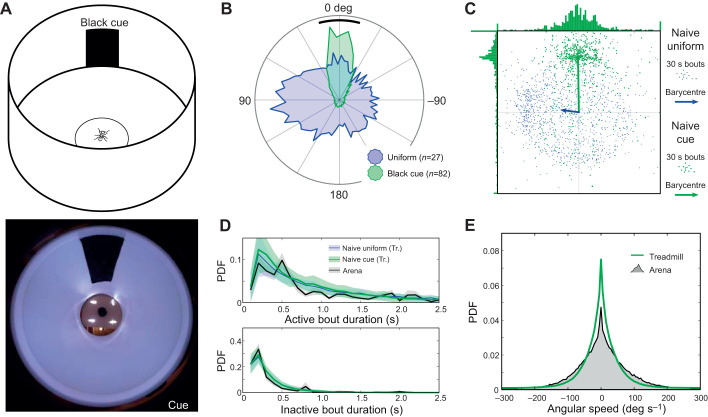
**Innate attraction toward a single conspicuous cue on the treadmill compared with arena behaviour.** (A) Innate response to a visual cue (a vertical black bar) was tested on the treadmill. The picture at the bottom shows the visual environment with the added cue from the ants' point of view on the servosphere. (B) Probability density function of the heading measured on the treadmill in a uniformly white environment (blue patch) or in the presence of a visual cue (green patch). (C) Mean resultant vector extracted from the experiment in a uniformly white environment (blue points) or in the presence of a cue (green points) on the treadmill. Each point represents the coordinate of the resulting vector for movement during 30 s intervals. The arrows indicate the overall resultant vectors for each group. The cue induced a clear and important bias over the *X*-axis and completely overwhelms the bias observed in the uniform white environment. (D) Distribution of active and inactive bout length during experiments on the treadmill with a single cue (green line) or in a uniformly environment (blue line) and in the arena with a corresponding cue (black line). Shading indicates ±s.e.m. (E) Distribution of angular speed in experiments with a single cue on the treadmill (green line) or in the arena (grey area).

In addition to checking for the resilience of the innate movement pattern, we looked at whether innate directed behaviour towards a single visual cue (Wallace, 1962; Voss, 1967; Wehner, 1972; Götz, 1987; Collett, 1988; Graham et al., 2003; Poehlmann et al., 2018 preprint; Giraldo et al., 2018; Green et al., 2019) is maintained on the treadmill. Ants were presented with a vertical black bar with an angular size of approximately 20 deg from the centre of the treadmill in a totally white environment (Fig. 3B). We clearly observed an oriented behaviour towards the cue for each individual when compared with the uniform environment (Fig. 5B). We also observed that the movement pattern of the ants, in terms of both active–inactive bouts and turning rate, was not drastically affected by the presence of the cue (Fig. 5CD, Fig. S1). However, the tendency to walk straighter than in a uniform environment (Fig. 4E) tends to diminish (Fig. 5E). Nevertheless, the global orientation toward the cue overrides the bias observed in the uniform environment (Fig. 5C).

### Transfer of learned behaviour

Our innate behaviour tests showed that ants maintained a relatively normal motor pattern on the servosphere despite any vibrations and the discrepancy in visual feedback implied by walking on the spot, but they did not allow us to check for the robustness of the motivation to express more complex behaviour such as foraging. To test this, we conducted two different experiments.

**Fig. 6.**
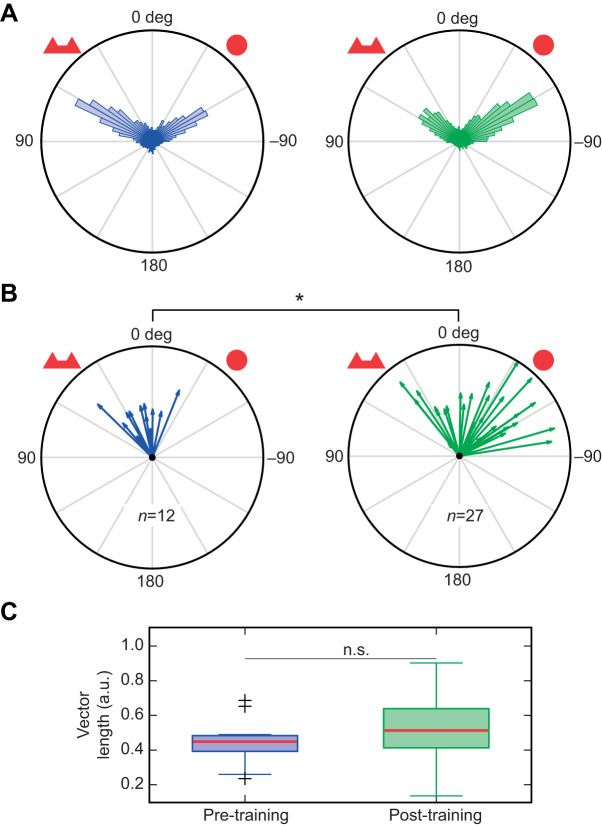
**Associative visual memory acquired during a T-maze conditioning is transferred onto the treadmill.** (A) Distribution of pooled heading data in both pre-tested (blue) and post-tested (green) ants. (B) Arrows indicate the orientation of the resultant mean vector of each experiment in pre-tested (blue) and post-tested (green) ants. Distribution of the vectors is significantly different between pre- and post-tested ants (Watson–Williams two-sample test, *n*_pre_=12, *n*_post_=27, *F*=9.56, *P*=0.0038). (C) Boxplots of the length of the resultant mean vector for each trial of both groups (no statistical significant difference between the groups; Kruskal–Wallis test, *n*_pre_=12, *n*_post_=27, χ^2^=1.34, *P*=0.2476). For each boxplot, the red line is the median value, the extremities of the box are the first and third quartile and the crosses indicate values treated as outliers, i.e. 1.5 times the interquartile range away from the median according to Matlab default method. *P<0.05; n.s., not significant.

We first used the ability of insects to associate a visual cue/pattern with reward (Guerrieri and d'Ettorre, 2010; Schwarz and Cheng, 2010, 2011; Giurfa, 2012) (Fig. 3A). Before any training, ants expressed a slight attraction to the two-triangles pattern [equivalent *t*-test, *n*=12, μ_pre_=15.04, 95% confidence interval (CI)=2.03, 28.05; Fig. 6A]. We therefore decided to reinforce the other cue during the training in a T-maze in order to better identify its effect on the choice of the ants on the treadmill afterwards. Indeed, after training, ants showed a clear shift toward the circle cue (Watson–Williams test, *n*_pre_=12, *n*_post_=27, *F*=9.56, *P*=0.0038, μ_post_=−21.06, 95% CI=–37.42, −4.69), suggesting a transfer of memory from the T-maze context, as well as the preservation of foraging motivation on the treadmill.

**Fig. 7.**
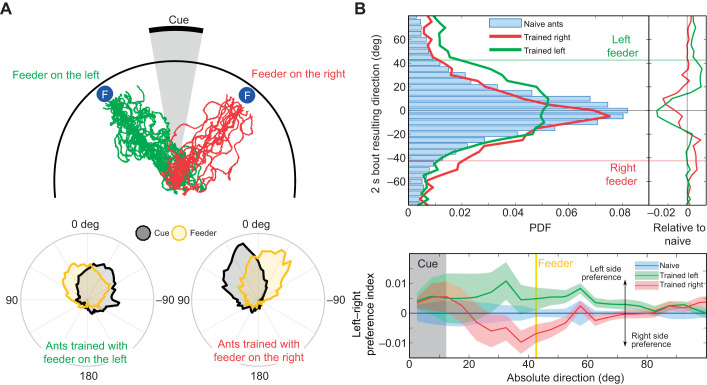
**Navigational memory of a feeder location acquired during arena conditioning is transferred onto the treadmill.** (A) Upper panel: paths of the selected ants during the last arena (120 cm in diameter) training before they were tested on the treadmill. Lower panels: distribution of both the visual cue and the feeder in the field of view of the ants trained with the feeder on the right or on the left of the cue during their last training session. (B) Upper panel: distribution of the orientation of the mean resultant vectors calculated for every 2 s bout during the first 5 min of each recording for naive ants (blue bars) or for trained ants with the feeder on the left (green line) or on the right (red line) of the cue. The right plot shows the same distributions for the two trained groups relative to the naive group distribution (naive distribution subtracted). Lower panel: left–right mean preference index for groups of ants trained with the feeder on the left (green) or on the right (red) of the cue. For each trial, the index is calculated by comparing the probability density function of the difference between opposite orientations for each absolute angle relative to the cue α: index(|α|)=*p*(α)–*p*(–α). A positive value indicates a preference for the left side of the cue and a negative value a preference for the right of the cue. The index calculated for the naive ants (blue shaded area) has been subtracted from each group of trained ants. Shaded areas indicate ±s.e.m.

**Fig. 8.**
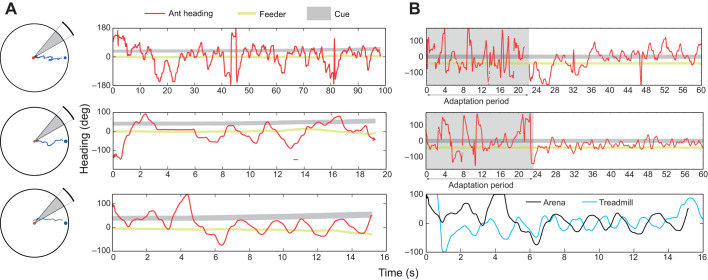
**Oscillatory heading behaviour in training ants is conserved from the arena to the treadmill.** (A) Examples of heading dynamics during the training in the arena (red trace). The black band shows the evolution of the orientation of the cue in regard to the ant's position along the path and the gold line the orientation of the feeder. On the left are shown the *X*,*Y* positions of the corresponding path. (B) The two first panels show examples of heading dynamics during the first 60 s on the treadmill of the corresponding ants. The last panel shows both heading dynamics in the arena (black line) and on the treadmill (blue line) during 16 s. The treadmill path has been selected starting just after the ≈20 s of adaptation observed generally (see panels above). Both signals have been centred arbitrarily to compare oscillation dynamics, but not normalized (i.e. the amplitude is conserved).

Going beyond simple association of a cue with reward, ants are able to learn the location of a feeder relative to distant visual cues (Durier et al., 2003). We assessed the ability of ants to reproduce such a navigational task on the treadmill by quantifying their angular orientation relative to a similar cue, after training them to reach a feeder displaced from a vertical bar in a circular arena of 120 cm diameter (Fig. 3B). To focus only on the transfer of the memory and not on the intrinsic ability of ants to perform the task, we first selected the ants that showed the best performance during training in the arena, by selecting those ants that showed fairly direct paths to the feeder for several training sessions in a row (Fig. 7A). Trained ants took less than 40 s to reach the feeder at the edge of the central platform (≍60 cm for a direct path). Note that the distribution of the feeder position in the visual field of the ants does not appear to reflect a process by which the visual cue is kept in a constant angular position, but rather that it oscillates between 0 and ±60 deg, depending on the training group (Fig. 7A). This is confirmed by a more detailed analysis of the dynamics of the ants' headings, which oscillate between the fixation of the feeder location (Fig. 8A) and the fixation of the cue, which appear to be the ‘limit’ of their oscillation.

When tested on the treadmill, trained ants oriented themselves rather more toward the cue than the feeder (Fig. 7B), although the training induced a significant effect in the direction of the virtual position of the feeder (Watson–Williams test, *n*_N_=87, *n*_L_=30, *n*_R_=15, *F*=6.65, *P*=0.0013). This trend was small but when compared with the naive distribution of orientations, a clear bump appeared around the location of the right or left feeder for each corresponding trained group (Fig. 7B). Furthermore, by calculating a left–right preference index (Fig. 7B) with regard to the cue location, we observed that the preference for the virtual feeder location was clearly determined by the training condition (either −42.5 or 42.5 deg from the centre of the cue). It is also noteworthy that in each group, the behaviour of the ants in the neighbourhood of the cue clearly expressed a preference for the right edge of the cue [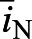
(α=12.5)=–0.01±0.004 (mean±s.e.m.); Wilcoxon test: 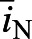
(12.5) versus 0, *z*=–2.7714, *P*=0.0056; 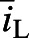
(12.5)=–0.006±0.007; 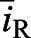
(12.5)=–0.005±0.005; where 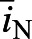, 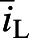, 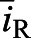 are the mean preference indices for the naive group, the group trained with the feeder on the left and the group trained with the feeder on the right, respectively; Fig. S6].

In addition to general direction statistics, we were also interested in the preservation of walking dynamics on the treadmill. It has previously been noted that wood ants approach a target cue by oscillating rather than fixating it (Collett et al., 2014), behaviour that we observed during the arena training sessions (Fig. 8A). On the treadmill, ants showed a similar oscillatory behaviour, which conserves consistent amplitude characteristics (Fig. 8B, lower panel; signals have been purposely centred but not normalized). In addition, we observed that ants showed an initial period of around 20 s in the two examples presented (Fig. 8B, upper panels; Fig. S5), during which the heading was more erratic, probably indicating a necessary delay for the ants to settle to this unfamiliar environment. The relative small duration of this adaptation and the preservation of the oscillatory behaviour support our claim that the treadmill can provide reliable unrestrained observation of wood ant behaviour.

**Fig. 9.**
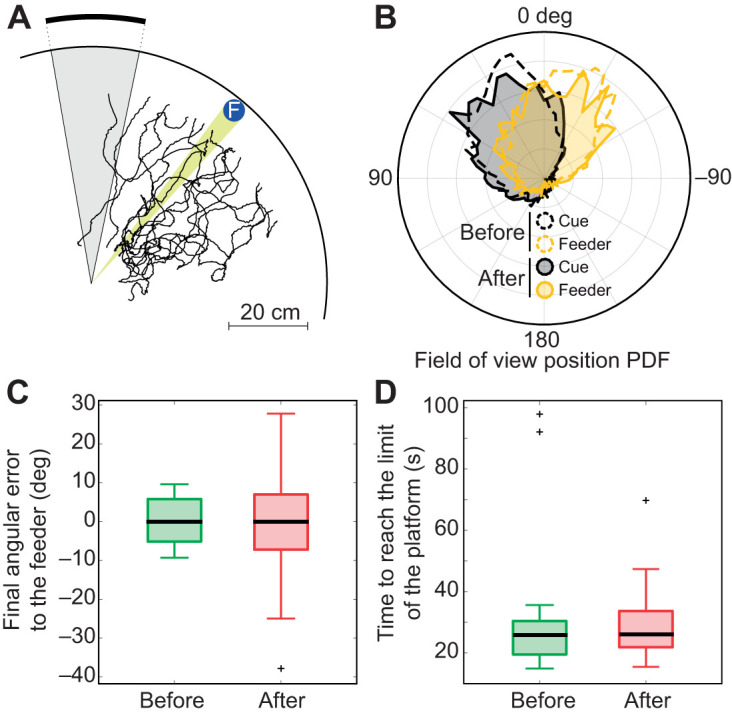
**Feeder location memory is preserved after experiencing long unrewarded experiments on the treadmill.** (A) Paths observed in the arena directly after the experiments conducted on the treadmill. (B) Probability density function of the orientation, relative to the ant's orientation, of the cue (black) and the feeder (yellow) before (dotted lines) and after (solid lines) the test on the treadmill. (C) Final angular error of the ant relative to the feeder calculated during the last learning session before (green) or the first learning session after (red) the test on the treadmill. (D) Time to reach the edge of the inner platform (estimated at 50 cm from the centre of the arena) calculated during the last learning session before (green) or the first learning session after (red) the test on the treadmill.

Finally, to complete our experiments, and because we are aiming at a device that allows interaction between classic arena/maze/field and VR experiments, we tested whether experiencing the treadmill would disturb memory or behaviour during retrieval experiments. At the very least, the total absence of reward during the trials on the treadmill could induce a reduction of the memory, or even reversal if this is experienced as punishing. To test this, we placed ants that had been tested on the treadmill back in the arena for an additional ‘training’ session. Fig. 9A shows the ants' path when experiencing again the cue in the arena. The directions taken to reach the feeder were clearly more noisy compared with that at the end of the training (Bartlett's test, *n*_before_=14, *n*_after_=14, *T*=13.893, *P*=0.0002; Fig. 7A,C), but still centred towards the feeder location (Kruskal–Wallis test, χ^2^=0.05, *P*=0.82; Fig. 7C), rather than attracted by the cue as we would expect from a return to naive state. Furthermore, the pattern of fixation of both the cue and the feeder location did not change drastically after unrewarded experiments on the treadmill (Fig. 9B) and the time taken to reach the edge of the platform did not seem to be much delayed [Wilcoxon signed-rank test (paired data), *n*_before_=14, *n*_after_=14, *Z*=0.2825, *P*=0.78; Fig. 9D], suggesting a straight path toward the edge of the platform and a maintained foraging motivation. These retrieval experiments were only conducted for ants from the group trained with the feeder on the right of the cue. Nevertheless, in the group trained with the feeder on the left, ants selected to be first tested on the treadmill were allowed to rejoin the training afterward and therefore could be selected several times. We were able to observe several ants that responded satisfactorily to the paradigm in the arena three times in a row (criterion to be tested again on the treadmill), indicating a maintenance of the foraging motivation as well as of the memory over sessions on the treadmill. This supports the paradigm of back-and-forth transfer between real arena experiments and VR treadmill experiments. Overall, it appears that the experience of very long (10 min) unrewarded trials on the treadmill in comparison to ≍40 s rewarded trials in the arena did not significantly extinguish the memory or the motivation of the ants.

## DISCUSSION

Our aim was to develop a means to study insect visual navigation in well-controlled but unrestrained walking conditions. To this end, we developed a treadmill (a 3-DOF servosphere) for wood ants (*F.* *rufa*) that compensated for their movement such that they remained in one location relative to their surroundings whilst freely walking untethered. Such treadmills have been employed previously in studies of insects with slow dynamics of movement (earlier work reviewed in Hedwig, 2017; more recent examples include Sakuma, 2002; Shigaki et al., 2016) but not for faster insects such as ants. We showed that using visual feedback control, the ant can be kept reliably within 5 mm of the apex of the sphere. We also showed that ants on the treadmill maintained walking dynamics similar to those of freely moving ants walking in an arena (Figs 4 and 5), and could transfer learnt visual associations between the two contexts (Figs 6 and 7).

### Advantages and disadvantages of the motion compensator

Many studies have used trackballs to investigate in detail how animals move in relation to a sensory stimulus such as an olfactory, auditory or visual cue (Schultheiss et al., 2017). These trackballs typically involve a sphere suspended on a cushion of air that is propelled by a tethered animal atop it (Van Swinderen, 2012; Moore et al., 2014; Paulk et al., 2014; Dahmen et al., 2017). This can set strong constraints on the sphere size, weight and shape, typically requiring bespoke manufacture of the system (Dahmen et al., 2017). A practical advantage of the motion compensator is that it can be built from commercially available components, and larger spheres (with lower curvature) can be used because the sphere is moved by motors, not the insect.

An additional advantage is that the motion compensator does not require a tether to be attached to the insect. This reduces or removes: (i) the effects of handling, (ii) the introduction of undesirable variability dependent on the expertise of the experimenter, and (iii) the potential change in motivation and walking dynamics caused by the attachment of a tether. It is known that handling of insects induces stress and the release of neuromodulators, which can alter behaviour long after the cause of the stress has ceased (Davenport and Evans, 1984; Harris and Woodring, 1992; Roeder, 1999). A recent study has also shown how aversive experiences can quickly affect the behaviour of ants (Wystrach et al., 2020). The diminished need for manipulation of the animal also facilitates the transition between naturalistic fieldwork and a fully controlled laboratory approach. Complex navigation experiments with social insects require the ability to return ants to their colony to feed their conspecifics in order to maintain their foraging motivation. Our apparatus allows a combination of field/arena/treadmill experiments to be used at different stages of a training programme, for example. In addition, behaviour persisted over long durations, over 2 h in some cases, and thus could support experiments more consistent with the long distances central foraging insects can cover.

Tethering an insect on a trackball can directly affect the motion of the animal (Spirito and Mushrush, 1979; Taylor et al., 2015) and it is often reported that tethered conditions quickly induce a drop in the insects' motivation. Instead, our setup offers the possibility to carry robustly large time scale experiments over 1 h, approaching ants' natural navigation. It is uncommon in the published literature on ants to find comparable duration on a classic trackball. To our knowledge, only Jensen and Holm-Jensen (1980) have observed similar bout lengths in wood ants, but in their experiments, the animals were forced to keep walking as the purpose was metabolic analysis. As a more recent comparison, Dahmen et al. (2017) tracked *Cataglyphis* desert ants on their tethered trackball for up to 7 min, but it is unclear whether this is the longest practical duration or whether some other experimental considerations determined this maximum. Nevertheless, our treadmill obviates the need for the physical constraints of a tether, allowing more naturalistic movement of the wood ants, as observed on a similar motion compensator for pillbug (Nagaya et al., 2017). Although some aspects of the motion compensation (such as vibration or altered motion feedback) could have altered the ants' behaviour, our results suggest that walking dynamics were not drastically impacted by the motion compensator. Comparison between ants on the treadmill and in an arena show little change in several aspects of walking such as the distribution of walking bout durations and the angular speed in a uniform environment and with a salient cue. In our experiments, behaviour persisted over long durations, even though walking on the spot is an open-loop situation and therefore should elicit some discrepancy in feedback, which could have led to a decrease in motivation. It still needs to be shown that additionally correcting for the ants' angular velocity to keep them in a fixed orientation, as can be done on the compensator (Shigaki et al., 2016), would not cause a similar decrease in behavioural motivation as seen in classic trackball experiments that prevent free rotation (Buatois et al., 2017).

The obvious disadvantage of not tethering the insect is that it prevents simultaneous intracellular neural recordings or imaging of brain activity, which is possible from head-fixed insects on trackballs. Nevertheless, extracellular recordings and optogenetic stimulation should remain viable and easier to achieve than in an arena owing to the limited movement distance. We also note that experiments in complete darkness are not possible, but the system should operate with lower light or infrared light (not visible to the insect), provided this is homogeneous, constant and has an adequate signal-to-noise ratio for the camera.

### Transfer of learnt behaviour on the motion compensator

Showing that the walking dynamics are not radically altered by the active motion compensation provided by our treadmill does not establish its suitability for navigation experiments. In particular, these require that the ant on the treadmill responds to presented stimuli in a manner consistent with its previous training experience off the treadmill. Therefore, we developed two different experiments to inspect the transfer of learnt tasks on the treadmill: an associative learning task and a proper navigation task.

Ants trained in a T-maze to associate a visual pattern to a food reward showed clear preference for that pattern when tested on the treadmill (Fig. 6). Transfer of learning in different contexts is a known ability in insects. It has particularly been shown in VR setups using associative learning paradigms (Buatois et al., 2017, 2018; Rusch et al., 2017). Here, it suggests that the recognition of certain visual cues can occur independently of the panoramic surrounding. Furthermore, our treadmill, by giving the ants the ability to freely scan the environment, allows a more detailed observation of active learning or recognition strategies (see Fig. 8). Recording orientation behaviour in the continuous manner offered by the treadmill (rather than at a single choice point) allows observation of persistence or suppression mechanisms in the memory process. In addition, long-duration observation provides a stronger statistical power to evaluate dichotomous and sequential choice in ants (Chameron et al., 1998; Schatz et al., 1999; Beugnon and Macquart, 2016).

Ants trained in an arena to approach a feeder displaced to one side of a visual cue (a vertical black bar) showed a tendency to orient their exploration toward the virtual position of the feeder (Fig. 7B) when tested on the treadmill. The transfer of some memory also supports the assumption that the foraging motivation is preserved on the treadmill, which is important with respect to further experiments on navigation behaviour. However, the shift in direction displayed on the treadmill was relatively weak. There may be several reasons for this. Firstly, the absence of any change in the size of the cue, and consequent discrepancy in the expected optic flow, could affect the behaviour (Brembs, 2009). Secondly, we observed that in the arena, orientation to the feeder did not occur immediately after release in the centre (Fig. 8A), suggesting that the cue could initially directly serve as a target (Graham et al., 2003). Therefore, our use on the treadmill of a cue with an angular size close to the cue observed from the centre of the arena may not have fully elicited the trained deviation towards the feeder. Thirdly, the strategy used by ants to reach the feeder in the arena is not to keep a constant heading towards the feeder location, but to oscillate around its orientation (Collett et al., 2014), and moreover, to use the cue as a limit of the oscillations (Fig. 8A). It is noteworthy that the exploratory oscillations are preserved on the treadmill. These preliminary observations suggest that, in future, systematic analysis of the ants' paths in a fully controlled closed-loop visual environment could be crucial to understand the active processes used by ants to inspect, memorize and recognize their paths.

A surprising result we observed during this experiment is the existence of a seemingly innate preference for the right edge of the cue (Fig. 7B). This right-edge preference occurs for ants trained with the feeder either on the right or on the left, suggesting a robust bias. This may be another line of evidence for lateralization in the insects' brain, which has received increasing attention in the last decade at both group/species (Kells and Goulson, 2001) and individual levels (Gaudry et al., 2013; Bell and Niven, 2014), particularly from an adaptive perspective (Niven and Frasnelli, 2018; Frasnelli, 2013). In wood ants, it has been observed in behaviour such as trophillaxis (Frasnelli et al., 2012) and memory encoding (David Fernandes and Niven, 2020).

### Potential applications of the treadmill

The existence of transfer of associative memory from classic T-maze to the motion compensator not only offers perspective to study memory processes but also provide an ideal benchmark to assess questions on upstream visual processes. The early processes at work in the visual pathways of ants have received little attention to date (Vowles, 1965; Cammaerts, 2008, 2013; Schwarz et al., 2011). Treadmills offer a tool for reverse engineering the visual abilities of ants in a more automated and large-scale approach than arena or maze experiments (Nagaya et al., 2017; Shigaki et al., 2016). Understanding feature extraction processes would be particularly crucial to incorporate in models that have been proposed to sustain visual memory of routes in ants, for example (Ardin et al., 2016).

An obvious requirement to facilitate such work is the addition of a naturalistic and closed-loop virtual environment to the treadmill set-up. VR systems have already been shown to be a realistic solution to studying insects vision in tethered (Dickinson, 2001; de Bivort and van Swinderen, 2016) or in arena conditions (Fry et al., 2009; Straw et al., 2010). The combination of a 3D virtual environment and the preservation of the naturalistic walking behaviour on a motion compensator should allow fully monitored training and experiments, accelerating our understanding of how insect navigation is controlled by the brain.
